# Developing Potential Endocrine Therapy Controlling Estradiol and DHT by Targeting 17β-HSD7 Against ER+ Breast Cancer

**DOI:** 10.3390/cells15151345

**Published:** 2026-07-27

**Authors:** Ruixuan Wang, Xiaoqiang Wang, Peng Su, Jenny Roy, Donald Poirier, Sheng-Xiang Lin

**Affiliations:** 1 Axe Endocrinology and Nephrology, CHU de Québec Research Center, Université Laval, Quebec City, QC G1V 4G2, Canada; ruixuan.wang.1@ulaval.ca (R.W.); peng.su.1@ulaval.ca (P.S.); jenny.roy@crchudequebec.ulaval.ca (J.R.); donald.poirier@crchudequebec.ulaval.ca (D.P.); 2 Department of Molecular Medicine, Faculty of Medicine, Université Laval, Quebec City, QC G1V 0A6, Canada; 3 Department of Cancer Biology & Molecular Medicine, Beckman Research Institute, City of Hope, 1500 E. Duarte Rd., Duarte, CA 91010, USA; xiaoerqd@gmail.com

**Keywords:** candidate compound for endocrine therapy against breast cancer, reductive 17β-hydroxysteroid dehydrogenases, dual control of sex hormones, feedback control on enzyme expression, cell cycle arrest, efficacy of 17β-HSD7 inhibitors on xenograft tumor shrinkage, cytotoxicity and basic short-term in vivo toxicity, hERG inhibition

## Abstract

Breast cancer (BC) is the most incidental cancer in women. Patients’ living conditions and survival rates have significantly improved with the success of two first-line endocrine therapies of selective estrogen-receptor modulators (SERMs from 1977) and aromatase inhibitors (AIs from 1995). Unfortunately, both therapies have produced significant resistance. AI resistance reaches 50% in metastatic estrogen-dependent BC cases, leading oncologists to seek a substitute for current therapies. We target the reductive 17β-hydroxysteroid dehydrogenase type 7 (17β-HSD7), which stimulates the synthesis of the active estrogen estradiol (E2) and reduces the potent androgen dihydrotestosterone (DHT) simultaneously, triggering negative feedback on 17β-HSD7 expression. INH7(464), an improved 17β-HSD7 inhibitor, efficiently blocks estrogen conversion at an IC_50_ of 92 ± 8 nM. INH7(464) inhibited cell proliferation by decreasing E2 levels and restoring DHT, successively arresting the cell cycle in G0/G1 phase. In vivo, INH7(464) reduced tumor size by 49% on Day 22 in BC xenografts with T47D. INH7(464) reduced E2 levels in mouse blood circulation and xenograft tumor tissue. There is no demonstrated cytotoxicity or cardiotoxicity of INH7(464) on cellular levels and mouse models under the experimental conditions. 17β-HSD7 is a potential target for estrogen-dependent BC therapy, while INH7(464) is promising for a preclinical lead compound (PCT/CA2022/050966).

## 1. Introduction

In 2022, breast cancer (BC) surpassed lung cancer as the most diagnosed and leading cause of cancer-related deaths among women, worldwide [1,2]. The primary subtype of BC is estrogen receptor positive (ER+), accounting for approximately 70% of BC cases [3]. Due to the established role of estrogens in the initiation and progression of BC, endocrine therapy has become a major treatment modality for this subtype. Two first-line endocrine therapies for ER+ BC include selective estrogen receptor modulators (SERMs), represented by tamoxifen, which block estrogen signaling, and aromatase inhibitors (AIs) such as letrozole, which inhibit endogenous estrogen biosynthesis [3].

Interestingly, the androgen receptor (AR) is expressed in more than 70% of primary and metastatic breast tumors [4,5]. AR is expressed in up to 90% of ERα-positive tumors [6]. Androgens were proven to inhibit estrogen-dependent tumor growth via AR mediation in the presence of estrogens at physiological concentrations [7]. Low concentrations of the potent androgen dihydrotestosterone (DHT), ranging from 0.01 nM to 1 nM, have demonstrated a surprising anti-proliferative effect on ER+ BC cells in the presence of physiological estrogen concentrations [8]. Both in vitro cell lines and patient-derived models have shown that AR acts as a tumor suppressor in ER+ BC [9]. Moreover, combining AR agonists with standard-of-care agents has improved therapeutic responses in ERα-positive BC, suggesting that low-level androgens, in the presence of estrogen, facilitate BC treatment [7]. These findings have led to a new concept in endocrine therapy that addresses the imbalance between estrogens and androgens, rather than focusing solely on estrogens [10]. Ongoing efforts, including preclinical and clinical studies, investigating AR agonists and AR antagonists for women with ER+ BC, highlight the urgent need for further study and development in this area.

Steroidogenic enzymes known as reductive human 17β-hydroxysteroid dehydrogenases (17β-HSDs) play a crucial role in sex steroid conversion, acting as critical regulators of the intracellular concentration of active sex steroids [11]. These enzymes catalyze the final step in the formation of all active androgens and estrogens and the initiation of their degradation [12]. The well-known estrogen synthase aromatase is a cytochrome P450 enzyme that plays a pivotal role in the conversion of androgens into estrogens, catalyzing the biosynthesis of estradiol and related estrogens [13,14]. Specifically, 17β-HSD7 plays a pivotal role in estradiol (E2) formation and has been found to convert dihydrotestosterone (DHT) into 5α-androstane-3β,17β-diol(3β-diol) [15,16]. Further analysis of clinical samples reveals that 17β-HSD7 is significantly overexpressed in breast tumor samples, showing a 2.50-fold expression (*p* < 0.0001) compared to surrounding normal tissue, based on statistical evaluation of public data in postmenopausal estrogen-receptor-positive BC [17]. In addition to its dual roles in estradiol activation and DHT inactivation, 17β-HSD7 expression is positively stimulated by E2, promoting E2 biosynthesis in BC cells [16,17,18]. The causal relationship of sex hormone regulation and the relative modification of cyclin D1, p21, Bcl-2, and Bik has been reported with the former INH7(10) inhibitor from which INH7(464) was developed [16]. Therefore, downregulation of 17β-HSD7 can potentially be achieved by blocking E2 [19]. Inhibiting 17β-HSD7 with the earlier INH7(80) inhibitor has shown a significant reduction of cell proliferation in hormone-receptor-positive BC cell lines MCF-7 and T47D [8,20]. Consequently, the dual-functional 17β-HSD7 emerges as a novel conceptual target for hormone-receptor-positive BC, and its inhibition can potentially shrink BC xenograft tumors [16].

This study aims to identify potential new therapeutics for ER+ BC using an improved 17β-HSD7 inhibitor (PCT/CA2022/050966 has now entered the national phase to target the ER and AR pathways, simultaneously). The new 17β-HSD7 inhibitor, INH7(464), was evaluated for its efficacy against BC and its in vitro cytotoxicity, and hERG channel safety, and basic short-term in vivo toxicity were studied. The results strongly demonstrate its potential as a promising drug candidate targeting a different protein from former endocrine therapeutic targets as a new lead compound against BC.

## 2. Material and Methods

### 2.1. Inhibitor Chemical Synthesis

The chemical structures of 17β-HSD7 inhibitors INH7(464) [21] and INH7(10) [22] are shown in Figure S1. All inhibitor stock solutions were prepared by dissolving the steroid in dimethyl sulfoxide (DMSO). Inhibitors were diluted to final concentrations with hormone-free medium for in vitro assays and with 0.4% methylcellulose aqueous solution and ethanol (0.4% MC: ethanol; 92:8 *v*/*v*) for in vivo assays.

### 2.2. Enzymatic Assay

#### 2.2.1. 17β-HSD 7 Inhibition Assays

**Figure 1:**
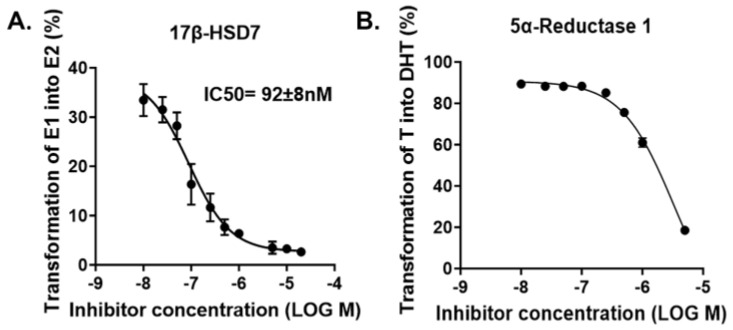
Inhibition of 17β-HSD7 and 5α-Reductase 1 by INH7(464). (**A**) IC_50_ determination of INH7(464) for the transformation of E1 to E2 by 17β-HSD7. The transformation was studied in quadruplicates at inhibitor concentrations ranging from 25 nM to 20 µM. (**B**) IC_50_ determination of INH7(464) for transforming T to DHT by 5α-Reductase 1. Transformation was tested at concentrations of the INH7 inhibitor from 10 nM to 5000 nM. Quadruple wells were used for each experimental condition and repeated independently in three experiments. Error bars represent the standard data deviation.

HEK-293 cells were transfected with pCMV-neo-h17β-HSD7 plasmids with the lipofectin transfection kit and grown under G-418 until resistant colonies were observed [22]. For IC50 value determination, HEK-293 cells overexpressing 17β-HSD7 were plated at a density of 5 × 10^5^ cells per well in 24-well plates with 3H labeled estrone (50 Ci/mmol) (Perkin-Elmer Life Sciences, Inc., Boston, MA, USA). The INH7(464) was added with concentrations ranging from 25 nM to 20 µM of 10 different concentrations. The medium was collected after 16 h of incubation. The culture medium was collected, and steroids were extracted twice with 2 mL diethyl ether. The steroids (E1 and E2) were separated by thin-layer chromatography (TLC) with a standard TLC protocol [22]. The % of transformation was calculated as follows: % Transformation (E1 to E2) = (3H) − E2/((3H) − E1 + (3H) − E2) × 100 (Figure 1A).

#### 2.2.2. 5α-Reductase 1 Inhibition Assays

For IC_50_ value determination, the HEK-293 overexpressing 5α-reductase 1 cells were plated at a density of 5 × 10^5^ cells per well in 24-well plates with ^14^C- labeled testosterone (100 µCi/mmol) (Perkin-Elmer Life Sciences, Inc. Boston, MA, USA). The INH7(464) inhibitor solution was added at concentrations ranging from 25 nM to 5 µM. The medium was collected after 16 h of incubation. The culture medium was collected, and steroids were extracted twice with 2 mL diethyl ether. The steroids (T and DHT) were separated by TLC with a standard TLC protocol [22]. The % of transformation was calculated as follows: % Transformation (T to DHT) = (^14^C) − DHT/((^14^C) − T + (^14^C) − DHT) × 100 (Figure 1B).

Each concentration used duplicate wells and was repeated in three independent experiments. The half maximal inhibitory concentration (IC_50_) values were calculated using Prism version 9.4.0.

### 2.3. In Vitro Assay

#### 2.3.1. Cell Culture

The BC cell line MCF-7 was grown in Dulbecco’s modified Eagle medium (DMEM) (Gibco, Life Technologies, Paisley, Scotland) with 10% fetal bovine serum (FBS) (Sigma, Oakville, ON, Canada). The BC cell line T47D cells were cultured in Roswell Park Memorial Institute (RPMI) 1640 medium (Gibco, Life Technologies, Paisley, Scotland) containing 10% FBS. The epithelial cell line MCF-10A, used as the control cell line, was grown in DMEM/F12 (Gibco, Life Technologies, Paisley, Scotland) with 5% horse serum (Gibco, Life Technologies, Paisley, Scotland). Human embryonic kidney (HEK)-293 cells were grown in minimum essential media (MEM) (Gibco, Life Technologies, Paisley, Scotland) with 500 µg/mL Geneticin™ Selective Antibiotic (G418) (Sigma, Oakville, ON, Canada). Dextran-coated charcoal-stripped (Sigma, St. Louis, MI, USA) FBS was used as a hormone-free (HF) culture medium. All cells were grown in a 5% CO_2_ atmosphere at 37 °C. All cell lines were purchased from the American Type Culture Collection (ATCC, Manassas, VA, USA).

#### 2.3.2. Inhibition of BC Cell Proliferation

The cell proliferation reagent WST-1 (Abcam, Cambridge, MA, USA) was used to measure BC cell proliferation changes. WST-1 is designed for the non-radioactive, spectrophotometric quantification of cell proliferation, growth, viability, and chemosensitivity in cell populations. The protocol was according to the manufacturer’s guide. The cells were plated at a density of 3 × 10^3^ cells per well in 96-well plates. Dehydroepiandrosterone (DHEA) and E1 (Sigma, St. Louis, MI, USA) were used as substrates. The culture medium was changed every 48 h. Each condition used quadruplicate wells and was repeated in three independent experiments.

#### 2.3.3. BC Cell Cycle Analysis

BC cells were plated at a density of 1 × 10^5^ cells per well in 6-well plates. Cells were treated with 2 µM INH7(464) with E1 or DHEA as substrate for 144 h. The medium was changed every 48 h. The cells were fixed with 70% ethanol and stained with propidium iodide (PI) (Molecular Probes, Invitrogen, Burlington, Canada) before analysis by flow cytometry using the BD LSR II (BD Bioscience, Milpitas, CA, USA). The results show the percentage of total cells in the G0/G1, S, and G2/M phases.

#### 2.3.4. Protein Extraction and Western Blot

The BC cells were treated with 2 µM INH7(464) for 144 h. Total proteins from BC cells were extracted by RIPA buffer (Invitrogen, Burlington, ON, Canada) with a 1% protease inhibitor cocktail (EMD Chemicals, Gibbstown, NJ, USA, 100:1 *v*/*v*). Total cell protein (50 µg) was separated on a 12% SDS-PAGE, then electroblotted onto polyvinylidene difluoride (PVDF) membranes (Amersham Hybond- PTM, GE Healthcare, Quebec, QC, Canada). The primary antibodies used were anti-17β-HSD7 (ab112006) (Abcam, Cambridge, MA, USA) 1:1000 and β-actin Loading Control Monoclonal Antibody (BA3R) (Invitrogen, Carlsbad, CA, USA) 1:5000. Mouse anti-rabbit IgG-HRP (sc-2357) (Santa Cruz Biotechnology, Santa Cruz, CA, USA) 1:5000 and m-IgGκ BP-HRP (sc-516102) (Santa Cruz Biotechnology, Santa Cruz, CA, USA) 1:5000 were used as secondary antibodies. Blots were visualized with Western Lighting Plus ECL (PerkinElmer, Waltham, MA, USA) enhanced chemiluminescence substrate for Western blotting, followed by exposure to X-ray films. The target band densities were quantified using the Image program (Molecular Dynamics, Sunnyvale, CA, USA). All samples were tested in triplicate and repeated in three independent experiments. The ratios between the target protein and corresponding β-actin were calculated to determine the relative protein expression.

#### 2.3.5. E2 and DHT Concentration

BC cells were plated in 6-well plates at a density of 1 × 10^5^ cells in duplicate wells and treated with 2 µM INH7(464) with the substrate E1 (0.1 nM). We determined the E2 and DHT levels in the culture supernatants immediately with the Estradiol EIA Kit (Cayman Chemical, Ann Arbor, MI, USA) and DHT ELISA Kit (Alpha Diagnostic International, San Antonio, TX, USA). The measurement and calculation were performed according to the manufacturer’s instructions and protocol. Data were reported as picomolar (pM). Each experiment was repeated three times.

#### 2.3.6. Cytotoxicity Assay

INH7(464) and the reference compound PTX were dissolved in DMSO and diluted at multiple concentrations with culture media to obtain the final desired concentration of 1 nM–100 µM. The incubation time after adding the compounds was 3 days. The half maximal effective concentration EC_50_ value represents the concentration that inhibits 50% of cell proliferation and was calculated using an iterative least square regression method by Prism version 9.4.0.

#### 2.3.7. The hERG Study

The evaluation of the effects of INH7(464) on the human potassium channel using HEK-293 cells transfected with a human ether-a-go-go-related gene (hERG) was performed by IPS Therapeutique (Sherbrooke, QC, Canada) [23]. The hERG inhibition assay was performed using a manual patch clamp to measure the amplitude of the currents produced by the hERG gene product. INH7(464) was tested at 0.1, 0.5, and 2 μM concentrations. The selective voltage-gated potassium channel blocker E-4031 (30 and 100 nM) were added to naive cells as a positive control. The hERG assay is used to assess the potential of the inhibitor to interfere with the rapidly activating delayed-rectifier potassium channel. The recordings were analyzed in Clampfit. A paired Student’s *t*-test with threshold for significance set at *p* ≤ 0.05 (n = 3) was performed.

### 2.4. Animals

The institutional animal ethics committee (Nagano, CHU de Québec-Université Laval, Québec, QC, Canada) approved the animal experiment protocols (see Institutional Review Board Statement at the end of the manuscript). All experiments were performed in the animal facility of CHU de Québec-Université Laval, which is approved by the Canadian Council on Animal Care (CCAC) and the Association for Assessment and Accreditation of Laboratory Animal Care. All treatments were performed using the s.c. mode.

#### 2.4.1. BC Xenograft Tumor Studies

The female nu/nu Br athymic mice (4 weeks) from Charles River, Inc. (Saint-Constant, QC, Canada) were ovariectomized (OVX) under isoflurane-induced anesthesia, and all mice were treated with E2 0.1 µg/0.05 mL (ethanol and propylene glycol; 8:92 *v/v*) s.c. injection 6 times per week per mouse after surgery. Three days after the surgery, 5 × 10^6^ MCF-7 cells or 1 × 10^7^ T-47D cells were suspended in a 0.1 mL mixture of 1:1 (*v*/*v*) Matrigel (BD), and culture medium was administered by s.c. injection in both flanks of each mouse.

The average tumor volume reached the experimental standard after 6 weeks of E2 injections. The MCF-7 or T47D group of mice with tumors was randomly split into 5 groups (15 mice per group): Group 1: E1 (0.1 µg) 6 times per week. Group 2: E2 (0.1 µg) 6 times per week per mouse. Group 3: Vehicle solution (DMSO and 0.4% methylcellulose; 8:92 *v/v*) 6 times per week per mouse. Group 4: INH7(10) (5 mg/kg) with E1 (0.1 µg) 6 times per week per mouse. Group 5: INH7(464) (5 mg/kg) with 0.1 µg E1 6 times per week per mouse. The inhibitors were dissolved in DMSO, and 0.4% methylcellulose was added to obtain a final 8% co-solvent concentration. The tumor volumes were measured twice a week with an external caliper. Two perpendicular diameters, the greatest longitudinal diameter (L) and the greatest transverse diameter (W), were recorded, and the tumor volumes were calculated using the modified ellipsoidal formula 0.52 × (L × W^2^). The volumes measured on the day before treatment were taken as 100%. The animals were euthanized after 3 weeks of treatment. The tumor specimens and estrogen-sensitive organs (uterus and vagina) were removed and weighed. The inhibitors were injected 3 h before euthanasia; blood samples were collected and frozen at −80 °C. Steroid quantification in plasma and different tissues was tested by gas chromatography tandem mass spectrometry (GC-MS) and performed by a metabolomics bioanalytical services platform (CHUL, Quebec, QC, Canada).

#### 2.4.2. Basic Short-Term In Vivo Toxicity Study

Female mice (Balb/c) were divided into 2 groups (8 mice per group): Group 1: Vehicle solution (DMSO and 0.4% methylcellulose; 8:92 *v/v*) administered weekly by s.c. injection for 3 consecutive weeks; Group 2: INH7(464) administered weekly by s.c. injection (20 mg/kg, 30 mg/kg, 60 mg/kg) for 3 consecutive weeks. INH7(464) was dissolved in DMSO, and 0.4% methylcellulose was added to obtain a final 8% co-solvent concentration. All female mice (Balb/c) were observed and weighed every day to notice any signs of toxicity (weight loss, inability to eat or to drink, abnormal posture, movement, or vocalization).

Animals were euthanized when tumor volume reached 1500 mm^3^, when tumors showed ulceration, or when the mice exhibited signs of severe distress, weight loss greater than 20%, or impaired mobility. All humane endpoints were established in accordance with ARRIVE 2.0 guidelines.

### 2.5. Statistical Analysis

Data are expressed as mean ± SEM. Statistical analyses were performed using GraphPad Prism 10. Normality was assessed using the Shapiro–Wilk test, and homogeneity of variance was evaluated before parametric analyses. For comparisons between two groups, an unpaired Student’s *t*-test was used when data were normally distributed with equal variance; otherwise, the Mann–Whitney U test was applied.

For comparisons among more than two groups, one-way ANOVA followed by Tukey’s multiple-comparison test was used. For experiments involving two independent variables, two-way ANOVA followed by Sidak’s multiple-comparison test was performed.

IC50 values from enzyme activity, cell proliferation studies, and cytotoxicity study were calculated by non-linear regression using a four-parameter logistic model. Cell cycle analysis, Western blot, sex steroid concentrations, and breast cancer xenograft tumor studies were analyzed using Student’s *t*-test or ANOVA as appropriate according to the number of groups.

Xenograft tumor-volume measurements were analyzed using repeated-measures two-way ANOVA or a mixed-effects model, with treatment group and time as fixed effects and individual animals as repeated measures. Multiple comparisons were adjusted using Tukey’s or Sidak’s post hoc tests as appropriate. The value of *p* ≤ 0.05 was considered statistically significant.

## 3. Results

The synthesis of the newly improved 17β-HSD7 inhibitor INH7(464) via structure–activity relationship (SAR) is reported in [21] and its structure comparison with former inhibitors is shown in Supplementary Figure S1.

### 3.1. Inhibition of E1 to E2 Activation by 17β-HSD7

To determine 17β-HSD7 activity inhibited by INH7(464), the enzymatic assays were performed in HEK-293 cells overexpressing 17β-HSD7 [22]. We tested the selected inhibitor INH7(464) for its ability to block the conversion of E1 into E2, the major action of 17β-HSD7 in steroidogenesis. Figure 1A shows that INH7(464) has a good inhibitory effect on 17β-HSD7 with an IC_50_ value of 92 ± 8 nM.

### 3.2. Inhibition of T Conversion into DHT by 5α-Reductase 1

To determine the selectivity of INH7(464) toward 5α-reductase 1 which is a short chain dehydrogenase like 17β-HSD7, we used HEK-293 cells expressing this enzyme. The IC_50_ value of INH7(464) is 3354 ± 346 nM for the conversion of T into DHT (Figure 1B), suggesting that INH7(464) has no effective inhibition effect on 5α-reductase 1.

### 3.3. Inhibitor INH7(464) Blocks BC Cell Proliferation and Arrested Cell Cycle in the G0/G1 Phase

**Figure 2:**
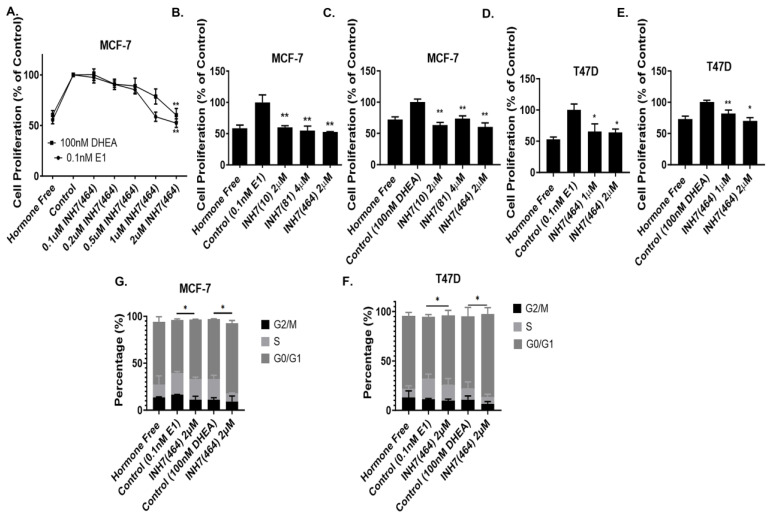
Illustration of the effect of INH7(464) on cell proliferation and cell cycle in BC cell lines. (**A**) Cell proliferation of MCF-7 treated with 1 × IC_50_ to 20 × IC_50_ of INH7(464) concentrations with 0.1 nM E1 or 100 nM DHEA. Data are presented as % of control (100%). (**B**,**C**) MCF-7 cell proliferation with various 17β-HSD 7 inhibitors, INH7(10), INH7(81), and INH7(464), over 144 h with 0.1 nM E1 or 100 nM DHEA. (**D**,**E**) T47D cell proliferation in the presence of 1 µM and 2 µM INH7(464) over 144 h with 0.1 nM E1 or 100 nM DHEA. (**F**) INH7(464) induced cell cycle arrest in G0/G1 phase in MCF-7 cells. The cell cycle was analyzed by flow cytometry after treatment with 2 µM INH7(464) in the presence of 0.1 nM E1 or 100 nM DHEA for 144 h; the treated group was compared with the control group. Data are shown as the percentage of total cells in the G0/G1, S, and G2/M phase. (**G**) INH7(464) induced cell cycle arrest in G0/G1 phase in T47D cells. The cell cycle was analyzed by flow cytometry with DAPI/ Triton X-100 solution. After treatment with 2 µM INH7(464) 144 h in the presence of 0.1 nM E1 or 100 nM DHEA, the treated group was compared with the control group. Data are shown as the percentage of total cells in G0/G1, S, and G2/M phases. Each sample was performed in triplicate and was repeated in three independent experiments. Error bars represent the standard deviation of the data. Statistical significance of the experimental results by *t*-test is shown by * *p* < 0.05; ** *p* < 0.01 vs. control.

INH7(464) displayed similar potency in both BC cell lines with an anti-proliferation effect. Based on the IC_50_ value (92 ± 8 nM) revealed in our study, the anti-proliferation effect of INH7(464) was tested with a concentration of 0.5 µM (5 × IC_50_), 1 µM (10 × IC_50_) and 2 µM (20 × IC_50_) in MCF-7 cells. A dose-dependent reduction in cell proliferation was observed in the presence of 0.1 nM E1 or 100 nM DHEA (Figure 2A). The cell growth was reduced by 14%, 41%, and 48% with 0.1 nM E1 and 11%, 21%, and 40% with 100 nM DHEA treated with 0.5 µM, 1 µM, and 2 µM INH7(464), respectively. Compared with previously studied 17β-HSD7 inhibitors, INH7(464) showed higher anti-proliferation efficacy. The cell growth decreased to 40% with 2 µM INH7(10) and 45% with 4 µM INH7 (81) in the presence of 0.1 nM E1 in MCF-7 cells, and the proliferation dropped 48% when treated with 2 µM INH7(464) (Figure 2B). In the 100 nM DHEA-induced MCF-7 cells, INH7(464) (2 µM) decreased cell proliferation by 40% compared with a 37% drop with 2 µM INH7(10) and 26% with 4 µM INH7(81) (Figure 2C). In fact, INH7(464) has an improved inhibitory effect on 17β-HSD7 with an IC_50_ value of 92 ± 8 nM, as compared to 195 ± 18 nM and 458 ± 38 nM for INH7(10) and INH7(81), respectively. In T47D cells, 1 µM INH7(464) reduced the cell growth by 35% with 0.1 nM E1 (Figure 2D) and by 18% with 100 nM DHEA (Figure 2E). Then 2 µM INH7(464) suppressed cell proliferation by 36% in the presence of 0.1 nM E1 (Figure 2D) and 30% with 100 nM DHEA (Figure 2E). INH7(464) showed similar efficacies in both BC cell lines (MCF-7 and T47D).

The flow cytometry assay was conducted to study INH7(464)’s effect on the cell cycle in both BC cell lines. The inhibitor arrested the cell cycle in the G0/G1 phase. In MCF-7, the cell cycle arrest in G0/G1 by INH7(464) increased by 12% in the presence of 0.1 nM E1 and 16% in the presence of 100 nM DHEA (Figure 2F). In T47D, the G0/G1 phase increased by 12% and 15% with 0.1 nM E1 and 100 nM DHEA, respectively, compared with the control (Figure 2G).

### 3.4. INH7(464) Inhibits E2 Formation, Restores DHT, and Decreases the 17β-HSD7 Expression in BC Cell Lines

**Figure 3:**
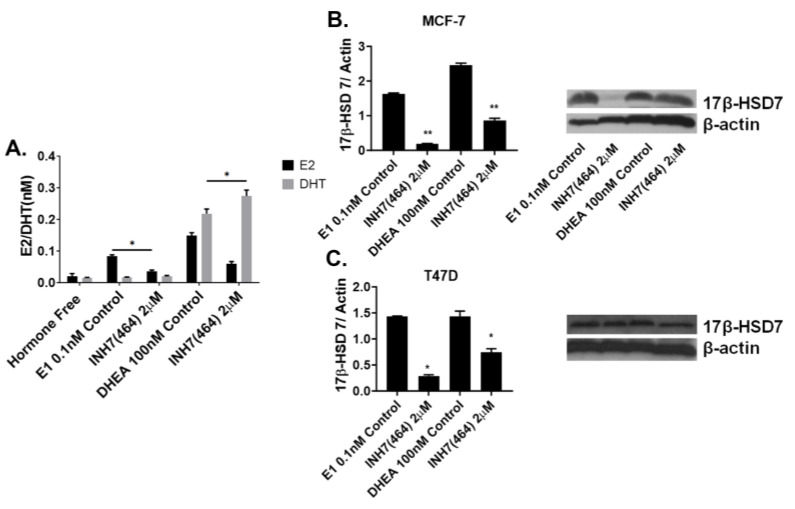
Illustration of the effect of INH7(464) on steroid conversion and 17β-HSD7 expression in BC cell lines. (**A**) E2/ DHT concentration in MCF-7 cells after treatment with INH7(464) 144 h. (**B**) The expression of target protein 17β-HSD7 after treatment with 2 µM INH7(464) for 144 h was determined by Western blot in MCF-7 cells. Total protein was extracted from MCF-7 cells. (**C**) The expression of target protein 17β-HSD7 after treatment with 2 µM INH7(464) for 144 h was determined by Western blot in T47D cells. Total protein was extracted from T47D cells. Each sample was performed in triplicate and was repeated in three independent experiments. Error bars represent standard data deviation. Statistical significance of the experimental results by *t*-test is shown by * *p* < 0.05; ** *p* < 0.01 vs. control.

17β-HSD7 inhibitor INH7(464) significantly blocked E2 formation and restored DHT concentration in MCF-7 cells (Figure 3A) in the presence of E1 (0.1 nM). INH7(464) reduced the E2 level by 57% and induced a 12% increase in DHT in the presence of 0.1 nM E1. Furthermore, following the provision of 100 nM DHEA as substrate, the E2 level dropped by 60%, and the DHT level increased by 26%. INH7(464) displays a significant effect on the blockage of E2 formation and an inhibitory effect on DHT inactivation. The expression of 17β-HSD7 in BC cells significantly decreased after 144 h of treatment with 2 µM INH7(464) due to E2’s effect on the endogenously expressed 17β-HSD7. Western blot results showed that the expression of 17β-HSD7 decreased 89 ± 0.4% in the presence of 0.1 nM E1 and 66 ± 1.8% in the presence of 100 nM DHEA in MCF-7 cells (Figure 3B), whereas the expression of 17β-HSD7 decreased 80 ± 1.6% with 0.1 nM E1 and 48 ± 8.6% with 100 nM DHEA in T47D cells too (Figure 3C).

### 3.5. Cytotoxicity Study for INH7(464)

**Figure 4:**
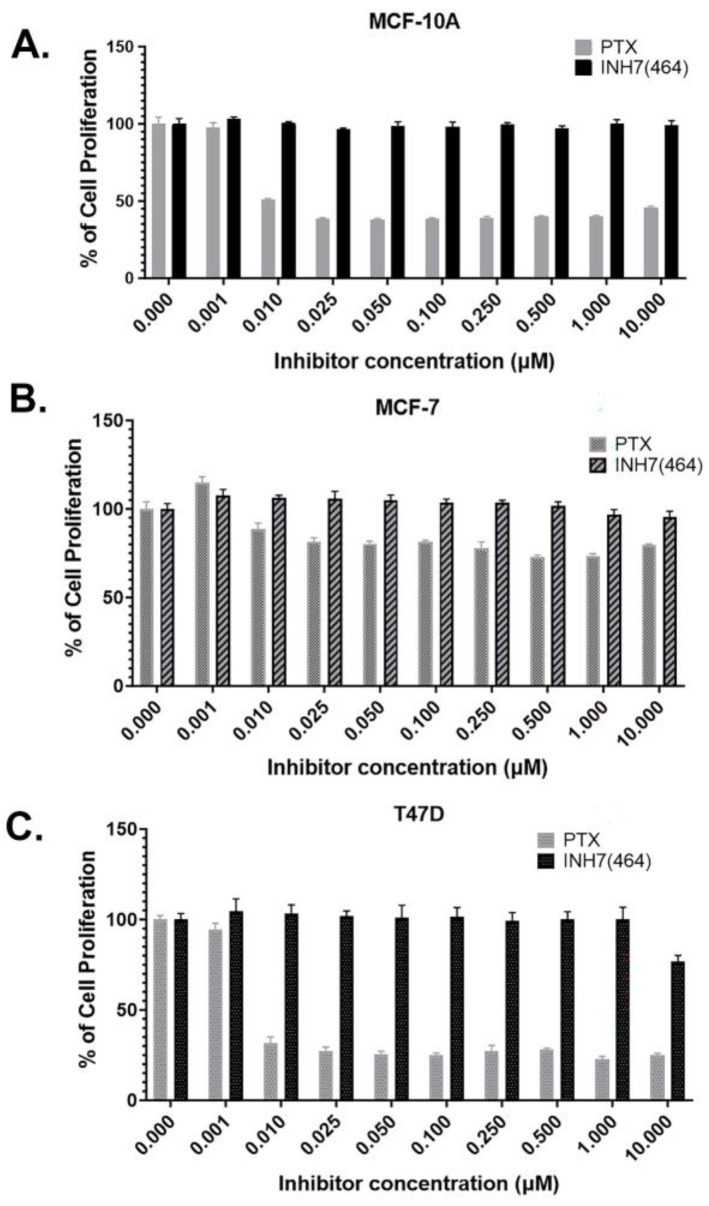
Illustration of the cytotoxic effect of INH7(464) and Paclitaxel (PTX) on Breast cells after 48 h of treatment at different concentrations. (**A**) Cytotoxic effect of INH7(464) and PTX on Breast epithelial MCF-10A cells. (**B**) Cytotoxic effect of INH7(464) and PTX on Breast cancer MCF-7 cells. (**C**) Cytotoxic effect of INH7(464) and PTX on Breast cancer T47D cells. Quadruple wells were used for each condition and repeated in three independent experiments. Each bar represents the mean of experiment results carried out in quadruplicate. Error bars represent standard data deviation.

The cytotoxicity of INH7(464) was compared to that of the reference compound paclitaxel (PTX) on the epithelial cell line MCF-10A, BC cell line MCF-7, and BC cell line T47D. The EC_50_ (half maximal effective concentration) values of INH7(464) were much higher than those of PTX in the three cell lines. In MCF-10A cells, at the range of concentrations tested (0.001 µM to 10 µM), it was impossible to determine the EC_50_ value of INH7(464), but the EC_50_ of PTX was 5.47 × 10^−4^ µM (Figure 4A). In MCF-7 cells, the EC_50_ of INH7(464) was 15.0 µM compared with 3.08 × 10^−3^ µM of PTX (Figure 4B). The EC_50_ value of INH7(464) was 30.0 µM, and the EC_50_ of PTX was 9.74 × 10^−3^ µM in T47D cells (Figure 4C). In comparison with PTX, INH7(464) exhibited weak anti-proliferative activity in breast cancer cell lines and non-detectable cytotoxicity toward non-tumorigenic epithelial cells within the tested concentration range.

### 3.6. In Vitro hERG Inhibition Testing with INH7(464)

The potential cardiotoxicity of the inhibitor candidate is an important filter for safe drug development and is usually evaluated in vitro by measuring the binding to the human cardiac voltage-gated potassium channel ether-a-go-go-related gene (hERG) [23]. The INH7(464) hERG study aimed at quantifying the effect of INH7(464) on the rapidly activating delayed-rectifier potassium selective current (IKr) generated under non-monic conditions in stably transfected HEK-293 cells. The concentrations 0.1, 0.5, and 2 μM of INH7(464) were selected for analysis. In vitro hERG inhibition testing with INH7(464) revealed a dose-dependent, maximal inhibition of 25% of the IKr current once corrected for vehicle and time-dependent run-down (Table 1). The maximal inhibition was observed at a free concentration of 0.5 µM, corresponding to free-circulating plasma levels. An IC_50_ value could not be calculated within the range of concentrations tested. The threshold for world regulatory concern is 50% inhibition of hERG. Preliminarily results support that INH7(464) is a safe inhibitor candidate. Inhibitions 36.6% and 73.2% of the current density were calculated for the positive control (E-4031) at 30 and 100 nM, respectively. The inhibition observed is in line with internal validation data generated in identical conditions and agrees with reported inhibition values for this compound. These results confirm the sensitivity of the test system to hERG-selective inhibitor E-4031. The study concludes that there is no potential cardiac risk associated with hERG inhibition due to exposure to INH7(464).

### 3.7. Inhibitor INH7(464) Induced E1-Stimulated BC Cell Xenograft Tumor Shrinkage

**Figure 5:**
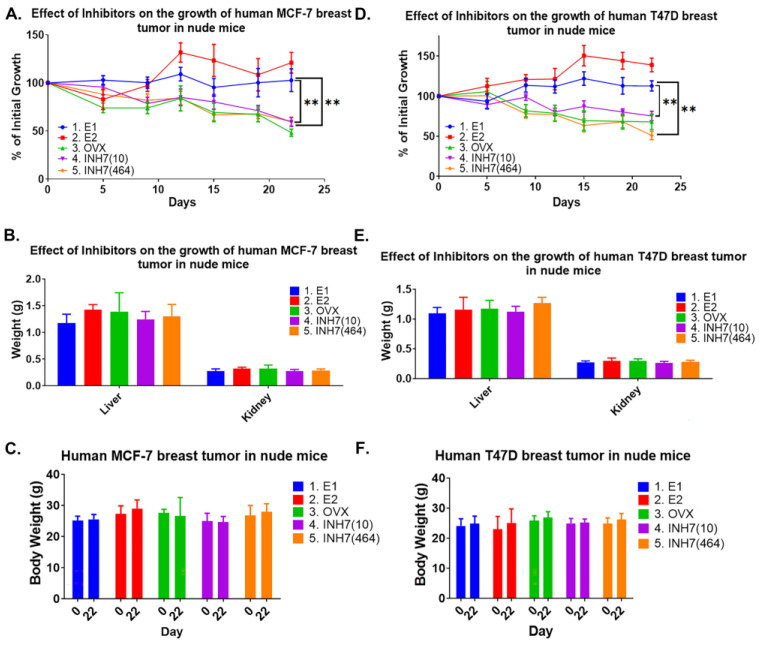
Illustration of the effect of 17β-HSD7 Inhibitors INH7(10) and INH7(464) on BC xenograft tumor studies. (**A**,**D**) Inhibitor INH7(464) induced E1-stimulated BC cell xenograft tumor shrinkage. (**B**,**E**) Liver and kidney weight of the mice in each group. Tissue was collected after 22 days of treatment. (**C**,**F**) Initial body weight (day 0) and final body weight (day 22) in each group. Each bar represents the mean of each group. Error bars represent standard data deviation. Statistical significance of experimental results by ANOVA test is shown by ** *p* < 0.01 vs. Control.

The effect of INH7(464) on tumor growth was investigated in the xenograft tumor model with BC MCF-7 or T47D cells in ovariectomized mice (Figure 5A,D). After being treated for three consecutive weeks, we observed that INH7(464) (5 mg/kg/day) given by subcutaneous (s.c.) injection effectively reduced MCF7 xenograft tumor growth (Figure 5A) to a level comparable to that of ovariectomized (OVX) mice unstimulated by E1. Examination of the results from the first two weeks revealed that INH7(464) induced MCF-7 tumor shrinkage from 100% (initial tumor volume) to 67% on Day 15 and that the tumor volume continued to decrease to 60% by the termination of treatment on Day 22 (*p* ≤ 0.001 vs. E1 group) (Figure 5A). INH7(10) reduced the MCF-7 tumor volume to 80% on Day 15 and 59% on Day 22 (*p* ≤ 0.001 vs. E1 group). The observation also showed T47D tumor shrinkage during treatment with INH7(464) or INH7(10) (Figure 5D). The inhibitor INH7(464) induced tumor reduction on Day 15 (64% of initial tumor size) and significant reduction on the last measurement (51%, *p* ≤ 0.001 vs. E1 group). INH7(10) also significantly affected T47D tumor growth (87% on Day 15 and 75% on Day 22).

No significant weight loss or gain from liver and kidney was detected between INH7-treated and untreated groups (Figure 5B,E). The body weights had no statistically significant difference between initial weight on Day 0 and final weight on Day 22 in each group (Figure 5C,F). The analysis of the behavior of the MCF-7 or T47D xenograft mice during the protocol or of their body weight did not show any sign of apparent toxicity of INH7(464) at 5 mg/kg/mouse/day.

### 3.8. Inhibitor INH7(464) Reduces E2 Levels in Plasma and Tumor Tissue

**Figure 6:**
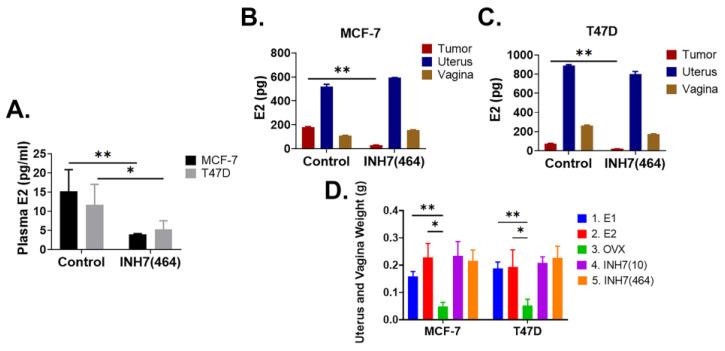
The inhibitor INH7(464) reduced E2 levels in plasma and tumors. (**A**) Xenographic mouse plasma E2 levels. (**B**,**C**) E2 levels in tumor and estrogen-sensitive organs (uterus and vagina) of mice. (**D**) Weight of estrogen-sensitive organs (uterus and vagina) in different groups. Each bar represents the mean of each group. Error bars represent standard data deviation. Statistical significance of experimental results by ANOVA test is shown by * *p* < 0.05; ** *p* < 0.01 vs. Control.

INH7(464) significantly reduced E2 levels in blood plasma and xenograft tumor tissue. In MCF-7 xenografts, plasma E2 levels decreased by 74% in INH7(464)-treated mice, while E2 levels in tumors dropped by 83% (Figure 6A,B). In T47D xenografts, the INH7(464)-treated group showed a 54% decrease in plasma E2 levels and 75% in tumor E2 levels (Figure 6A,C). The E2 levels in estrogen-sensitive uterus and vagina showed no significant difference between INH7-treated groups and E1 control groups in both MCF-7 and T47D xenografts (Figure 6B,C). The significant E2 level declines only in tumors, not in other tissues, demonstrating that INH7(464) inhibits only human 17β-HSD7 in xenograft tumors whose structure is significantly different from the mouse enzyme. INH7(464) only targets human 17β-HSD7 in xenograft tumors without affecting the mouse 17β-HSD7 in other organs. The weights of estrogen-sensitive uterus and vagina are also shown in Figure 6D. There was no significant difference in uterus and vagina weight observed between the INH7-treated groups and the E1 control group in both the MCF-7 and T47D xenografts. But the uterus and vagina weight increased 3.2-fold with 0.1 µg E1 daily and 4.6-fold with 0.1 µg E2 daily vs. OVX group in MCF-7 xenograft mice. The presence or absence of estrogens produces this difference. T47D xenograft mice also yielded similar results in estrogen organs; the weight increased 3.6-fold with 0.1 µg E1 daily and 3.7-fold with 0.1 µg E2 daily vs. OVX group. These results suggest that the human INH7 modulated circulating sex steroid hormones without a systemic inhibitory effect on estrogen-sensitive mouse organs under the experimental conditions using a dose of 0.1 µg E1 daily.

### 3.9. High-Dose INH7(464) Toxicity Study in Mice

**Figure 7:**
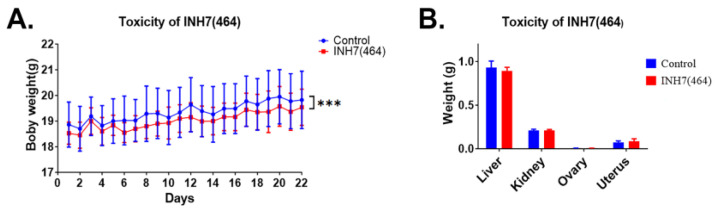
Illustration of the toxicity study of high doses of the INH7(464) inhibitor in mice. (**A**) Body weight of the mice in control and INH7(464)-treated groups. Statistical significance of experimental results by ANOVA test is shown by *** *p* < 0.001 vs. Control. (**B**) Liver, kidney, and estrogen-sensitive organ (ovary and uterus) weight in the control and INH7(464)-treated groups.

Increasing doses of INH7(464) were given in successive weeks, and all mice were carefully monitored during the week for toxicity signs approved by the ethics committee. No sign of apparent toxicity was noted for each dose of INH7(464) (20 mg/kg, 30 mg/kg, 60 mg/kg) given by s.c. injection for 3 consecutive weeks. The body weights in the control and INH7(464) groups were both stably increasing during the 3 consecutive weeks (Figure 7A). There was no significant difference in the weight of the liver, kidney, and estrogen-sensitive organs (ovary and uterus) between controls and data points in the INH7(464) group (Figure 7B).

## 4. Discussion

Patients with hormone receptor-positive BC require endocrine treatment as the standard of care for 5–10 years [24]. Aromatase inhibitors (AIs), such as letrozole, are competitive inhibitors of the aromatase enzyme, which reduces estrogen synthesis, and are primarily used in postmenopausal women [25]. Tamoxifen, the most widely used selective estrogen receptor modulator (SERM), is recommended for premenopausal patients as the first generation of endocrine treatment [25,26]. The main strategy for endocrine therapy in BC involves blocking estrogen’s action on ERα. However, this treatment is often limited by the development of resistance [26].

Studies have shown that up to 40–50% of patients with hormone-receptor-positive BC who are treated with adjuvant endocrine therapy will eventually relapse [27]. Resistance to endocrine therapy is the most significant challenge in treating hormone-receptor-positive BCs. In fact, these patients can be at risk of recurrence even after 10 years [28,29]. Despite extensive studies on the role of sex steroid conversion in BC over the past 2 to 3 decades, fundamental progress in endocrine therapy has been limited. Therefore, drugs that target different signaling pathways and proteins, representing a potential approach to endocrine therapy, could offer a novel conceptual treatment to overcome endocrine resistance [26].

Most women develop breast cancer during their postmenopausal years [24,30]. After menopause, the ovaries stop secreting estradiol, making DHEA, a precursor steroid of adrenal origin, the sole source of all estrogens and androgens in postmenopausal women [31]. Intracrine steroidogenic and steroid-metabolizing enzymes can then convert the high-circulating levels of DHEA into active sex steroids within peripheral tissues [12]. These enzymes have thus become potential targets for therapy in hormone-receptor-positive breast cancer [11,32]. A study of steroid-converting enzymes in 1097 clinical samples found that 17β-HSD7 is significantly upregulated in estrogen-receptor-positive breast cancer in postmenopausal women. This statistical evaluation supports 17β-HSD7 as a potential novel target for endocrine breast cancer treatment [17], due to its dual role in regulating estrogens and androgens, along with its feedback mechanism that enhances its expression through estrogen activation.

Several studies have indicated that the potential effect of androgen DHT is to facilitate BC treatment. DHT effectively inhibited BC cell viability, migration, and invasion in cellular models [33] and xenograft nude mice models [34], even in the presence of physiological concentrations of estradiol (E2). An in vivo study of estrogen-dependent BC found that the inhibitor INH7(10) could shrink tumors while decreasing E2 levels and increasing DHT levels in plasma [16].

Our study demonstrated that the 17β-HSD7 inhibitor INH7(464) reduced cell proliferation by up to 48%, arrested the cell cycle in the G0/G1 phase in the two hormone-receptor-positive BC cell lines MCF-7 and T47D, and induced a 49% shrinkage in xenograft tumors. Among the evaluated 17β-HSD7 inhibitors, INH7(464) demonstrated the lowest IC_50_ value (92 ± 8 nM). In comparison, INH7(10) (compound EB-357-030) exhibited an IC_50_ of 195 ± 18 nM, approximately twofold higher, whereas INH7(81) (compound 81) showed a markedly weaker inhibitory potency with an IC_50_ of 458 ± 38 nM [16,21], about fivefold that of INH7(464). The synthesis of a group of 17β-HSD7 inhibitors, as the first generation, was reported in Bellavance et al. in 2009 [22]. In vitro and in vivo studies for INH7(10) were reported in 2015 [16].

Targeting cell cycle pathways has emerged as a crucial opportunity for therapeutic intervention in overcoming endocrine-resistant breast tumors [35,36,37]. In terms of sex steroid conversion, INH7(464) reduced E2 levels by 60% and increased DHT levels by 26% compared to control groups in BC cell lines. Additionally, protein levels of 17β-HSD7 were downregulated due to the inhibition of 17β-HSD7. In BC cells, E2 can stimulate the recruitment and DNA binding of nuclear factor 1 to the promoter region of the HSD17B7 (17β-HSD7) gene, thereby increasing the expression of 17β-HSD7 [19]. It is essential for endocrine therapy to balance benefits against potential toxicity and impaired quality of life. High doses of INH7(464) showed no potential cardiotoxicity at the cellular level and no basic short-term toxicity in vivo, indicating it as a potentially safe drug candidate.

**Figure 8:**
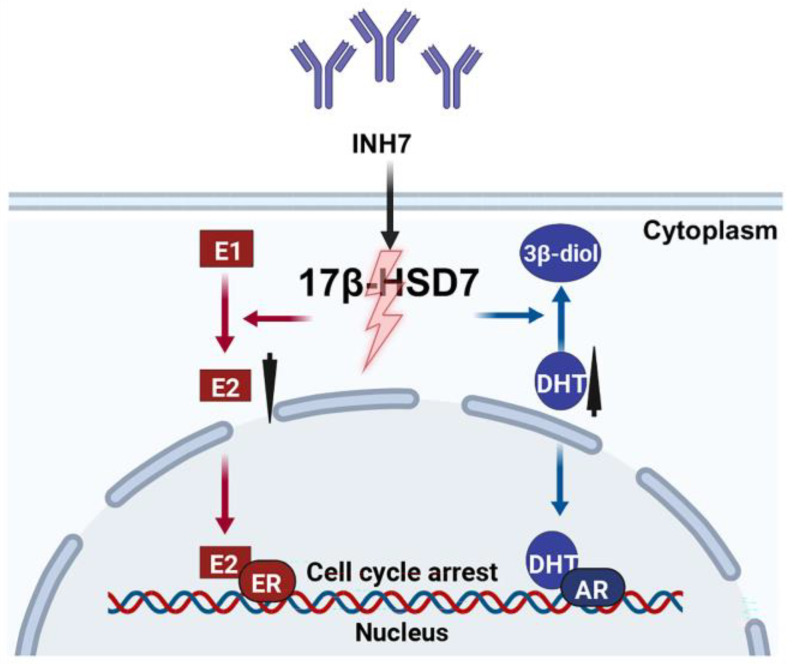
Schematic representation of the mechanisms of 17β-HSD7 inhibition in breast cancer cells. Inhibition of 17β-HSD7 suppresses BC cell proliferation and induces cell-cycle arrest by reducing E2 production and restoring DHT levels. Red arrows indicate the estrogen metabolic/signaling pathway, blue arrows indicate the androgen metabolic/signaling pathway, black arrows indicate the action of INH7 and changes in sex steroid levels, and the red lightning symbol indicates inhibition of 17β-HSD7. The figure was created with BioRender.com.

Importantly, INH7(464) exhibited the lowest IC_50_ value among the tested inhibitors, indicating a markedly improved biochemical potency against 17β-HSD7. Beyond its enzymatic inhibition, INH7(464) significantly affected steroid hormone balance in breast cancer cells. The consistent biochemical potency, the modulation of estrogen–androgen balance, and the observed suppression of cell growth both in vitro and in vivo support the therapeutic potential of INH7(464). These findings further highlight the reductive 17β-HSD type 7 as a promising target candidate for endocrine therapy in hormone-receptor-positive BC, where inhibition of 17β-HSD7 can slow tumor growth and induce cell cycle arrest through the combined reduction of E2 formation and restoration of DHT levels (Figure 8).

We also carried out computational docking to study the selectivity of INH7(464). Enzyme crystal structures for aromatase were directly downloaded from the Protein Data Bank (https://www.rcsb.org/, PDBID: 3S7S, 3EQM, accessed on 28 June 2026) for computational docking. The ligand, ions and waters of the crystal structure were removed before docking. GOLD software (Hermes 2026.1.0 (Build 483767)) was used for docking analysis of all compounds. An amount of 10 Å around the original ligand was set as the docking site. For each ligand, a maximum of 10 structures were generated, and the ChemPLP fitness was computed. ChemPLP uses the ChemScore hydrogen bonding term and multiple linear potentials to model van der Waals and repulsive terms. Higher ChemPLP scores are indicative of superior docking results. Protein–ligand binding affinity was calculated by PRODIGY (https://wenmr.science.uu.nl/prodigy/).

The PLP fitness score for INH7(464) docked into the structure of aromatase (3S7S), another critical enzyme for estradiol synthesis, is 56.29 which is much weaker than the aromatase inhibitor exemestane docked into this aromatase structure. The latter yielded a fitness score at 89.98. Very similarly, INH7(464) docked into the aromatase structure (3EQM) has a fitness score at 51.57 which is much weaker than androstenedione docking with a fitness score at 90.8.

Our findings show that INH7(464) inhibits BC development both in vitro and in vivo. We propose INH7(464) as a potential lead compound against hormone-receptor-positive BC, due to its dual regulation of estrogens and androgens and modulation of the cell cycle. INH7(464) is an evolution of the first-generation inhibitors with significantly lower IC_50_, and is the only inhibitor studied in in vitro and basic in vivo experiments reaching PCT. Further pharmacokinetic and oral administration studies are underway. Thus, following the completion of preclinical studies, this inhibitor is poised to become an investigational new drug for ER+ BC, with potential to become a new endocrine therapy, if clinical trials succeed.

## Figures and Tables

**Table 1:** Effect of inhibitors on hERG Current Density from Transfected HEK 293 Cells at 20 mV.

	Corrected Normalized Current Density
Baseline	1.000
INH7(464), 0.1 μM	0.937
INH7(464), 0.5 μM	0.750
INH7(464), 2 μM	0.788
E-4031, 30 nM	0.634
E-4031, 100 nM	0.268
Washout	0.715

## Data Availability

The data supporting the conclusions of this article are included in the article/Supplementary Material. Additional raw data, including flow cytometry FCS files, uncropped Western blot images and the datasets used for statistical analyses, will be made available by us upon reasonable request, without undue reservation.

## Supplementary Materials

The following supporting information can be downloaded at: https://www.mdpi.com/article/10.3390/cells15151345/s1. The supplementary materials include Figure S1 and Supplementary Methods.

## Abbreviations

17β-HSD7	17β-hydroxysteroid dehydrogenase type 7
AI	aromatase inhibitor
AR	androgen receptor
BC	breast cancer
CCAC	Canadian Council on Animal Care
DHT	dihydrotestosterone
DMSO	dimethyl sulfoxide
DHEA	dehydroepiandrosterone
DMEM	Dulbecco’s modified Eagle medium
E1	estrone
E2	estradiol
EC50	half maximal effective concentration
ER+	estrogen receptor positive
FBS	fetal bovine serum
GC-MS	gas chromatography tandem mass spectrometry
HEK	human embryonic kidney
hERG	ether-a-go-go-related gene
HF	hormone-free
IC50	half maximal inhibitory concentration
MC	methylcellulose aqueous
MEM	minimum essential media
OVX	ovariectomized
PI	propidium iodide
pM	picomolar
PTX	paclitaxel
PVDF	polyvinylidene difluoride
RPMI	Roswell Park Memorial Institute
SERM	selective estrogen-receptor modulator
T	testosterone
TLC	thin-layer chromatography
