# Supplementary material for: Developing Potential Endocrine Therapy Controlling Estradiol and DHT by Targeting 17β-HSD7 Against ER+ Breast Cancer
Source: Cells. 2026 Jul 27;15(15):1345. doi: 10.3390/cells15151345 (PMC13465700; doi:10.3390/cells15151345)
Supplement: Supplementary file 1 [file cells-15-01345-s001.zip › cells-4342177-supplementary.pdf]

Supplementary Methods

Randomization

After xenograft establishment, mice were randomly assigned to the experimental groups using the randomization function in Microsoft Excel. A random number was generated for each animal, and animals were allocated to treatment groups according to the randomized order. Randomization was performed after tumors reached the predefined treatment initiation volume to ensure comparable baseline tumor sizes among the treatment groups.

Blinding

Blinding was not implemented during treatment allocation or tumor measurements because the same investigator was responsible for drug administration and longitudinal monitoring of tumor growth throughout the study. This approach was adopted to maintain consistency in treatment administration and tumor measurements.

Sample Size Justification

The sample size (n = 15 mice per group) was determined based on our previous experience using the same xenograft model. In our previous study, 10 mice per group were sufficient to evaluate treatment efficacy. In the present study, five additional animals per group were included to account for potential attrition during the experimental period and to ensure adequate numbers of animals for the predefined statistical analyses without requiring replacement of animals after study initiation. No animals were lost during the experiment, and all 15 mice in each group were included in the final analyses.

Inclusion and Exclusion Criteria

Only mice whose xenograft tumors reached the predefined treatment initiation volume of approximately 100 mm^3 were eligible for randomization. Animals whose tumors failed to reach this predefined volume before treatment initiation were excluded prior to randomization. After randomization, no animals or data points were excluded, and all randomized animals were included in the final statistical analyses.

Humane Endpoints

Humane endpoints were predefined in accordance with institutional animal welfare regulations and the ARRIVE guidelines. Animals were monitored regularly throughout the study and were euthanized if they exhibited signs of pain or distress, excessive tumor

burden, significant body weight loss, or impaired normal behavior. No animals reached the predefined humane endpoints before the scheduled experimental endpoint.

Statistical Analysis

Data are presented as the mean ± SEM. Statistical analyses were performed using GraphPad Prism 10 (GraphPad Software, La Jolla, CA, USA). Data normality was assessed using the Shapiro–Wilk test, and homogeneity of variance was evaluated before parametric analyses. For comparisons between two groups, an unpaired Student's t-test was used for normally distributed data with equal variances; otherwise, the Mann–Whitney U test was applied. For comparisons among more than two groups, one-way ANOVA followed by Tukey's multiple-comparison test was performed. Experiments involving two independent variables were analyzed using two-way ANOVA followed by Sidak's multiple-comparison test. IC50 values for enzyme activity assays, cell proliferation assays, and cytotoxicity assays were calculated by nonlinear regression using a four-parameter logistic model. Cell-cycle analysis, Western blot quantification, sex steroid concentration measurements, and other endpoint analyses were analyzed using Student's t-test or one-way ANOVA, as appropriate, according to the number of groups. For breast cancer xenograft studies, longitudinal tumor-volume measurements were analyzed using repeated-measures two-way ANOVA or a mixed-effects model, with treatment group and time as fixed effects and individual animals treated as repeated measures. Multiple comparisons were adjusted using Tukey's or Sidak's post hoc tests, as appropriate. A two-sided P value ≤ 0.05 was considered statistically significant.

Supplementary Figure

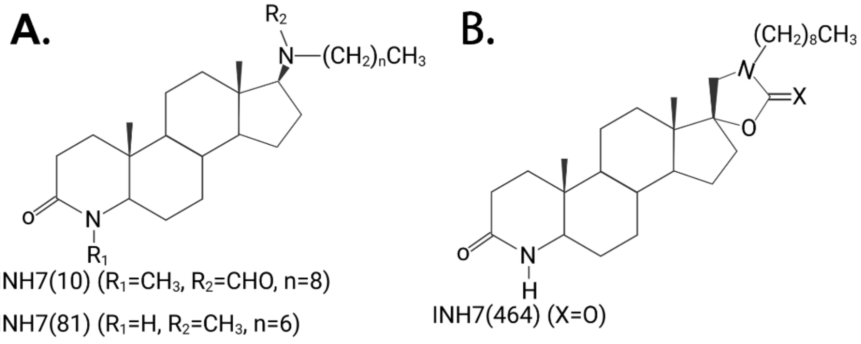

Supplement Figure S1
